# HS-BAμE: A New Alternative Approach for VOCs Analysis—Application for Monitoring Biogenic Emissions from Tree Species

**DOI:** 10.3390/molecules28031179

**Published:** 2023-01-25

**Authors:** Oriana C. Gonçalves, Jéssica S. R. F. Cerqueira, Ana S. Mestre, Nuno R. Neng, José M. F. Nogueira

**Affiliations:** Centro de Química Estrutural, Institute of Molecular Sciences, Departamento de Química e Bioquímica, Faculdade de Ciências, Universidade de Lisboa, 1749-016 Lisboa, Portugal

**Keywords:** BVOCs, HS-BAμE, HS-SPME, GC-MS, *Eucalyptus globulus* Labill., *Pinus pinaster* Aiton

## Abstract

In this work, a new analytical approach is proposed for monitoring biogenic volatile organic compounds (BVOCs) by combining headspace bar adsorptive microextraction (HS-BAμE) with gas chromatography–mass spectrometry (GC-MS). The HS-BAμE methodology was developed, optimized, validated and applied for the analysis of BVOCs emitted from two tree species (*Eucalyptus globulus* Labill. and *Pinus pinaster* Aiton) and compared with headspace solid phase microextraction (HS-SPME), commonly accepted as a reference technique. To achieve optimum experimental conditions, numerous assays were carried out by both methodologies, studying the release of the five major monoterpenoids (α-pinene, β-pinene, myrcene, limonene and 1,8-cineole) from the leaves of the tree species, whereas the maximum selectivity and efficiency were obtained using an activated carbon and PDMS/DVB fiber as sorbent phases for HS-BAμE and HS-SPME, respectively. Under optimized experimental conditions, both methodologies showed similar profiling and proportional responses, although the latter present a higher sensitivity in the analytical configuration used. For the five monoterpenoids studied, acceptable detection limits (LODs = 5.0 μg L^−1^) and suitable linear dynamic ranges (20.0–100.0 mg L^−1^; *r*^2^ ≥ 0.9959) were achieved, and intra- and inter-day studies proved that both methodologies exhibited good results (RSD and %RE ≤ 19.9%), which indicates a good fit for the assessment of BVOCs by the HS-BAμE/GC-MS methodology. Assays performed on sampled leaves by both optimized and validated methodologies showed high levels of the five major BVOCs released from *E. globulus* Labill. (10.2 ± 1.3 to 7828.0 ± 40.0 μg g^−1^) and *P. pinaster* Aiton (9.2 ± 1.4 to 3503.8 ± 396.3 μg g^−1^), which might act as potential fuel during forest fire’s propagation, particularly under extreme atmospheric conditions. This is the first time that BAμE technology was applied in the HS sampling mode, and, in addition to other advantages, it has proven to be an effective and promising analytical alternative for monitoring VOCs, given its great simplicity, easy handling and low cost.

## 1. Introduction

Volatile organic compounds (VOCs) are emitted as gases from several anthropogenic (e.g., fossil fuels) but also natural (e.g., plants, animals, etc.) sources, are characterized to have high vapor pressures at room temperature, i.e., low boiling points (50 to 250 °C), and include several classes of semi-volatile compounds. Some are responsible for the odour of scents, largely emitted by plants, usually referred to as ‘biogenic volatile organic compounds’ (BVOCs), where the most common classes are terpenoids. BVOCs emissions are affected by a variety of factors, such as the temperature, which determines the rates of volatilization and growth, and sunlight, which regulates the biosynthesis that occurs almost exclusively into the stomata of the leaves. Recently, studies have shown the plant’s capacity for producing highly reactive and flammable BVOCs when heated, which can lead to outbreaks of long-distance fires. As these BVOCs have densities lower than that of air, it has been suggested that the emitted fraction, composed mainly of terpenoids, might accumulate at lower altitudes and under vegetation [1]. Thus, in the presence of certain weather conditions and other combustion gases, the accumulated terpenoids can cause a rapid and intense ignition, increasing the flame height and accelerating the fire spread [2]. Therefore, it is of utmost importance to study the type, number and chemical characteristics of the BVOCs emitted from the main tree species, particularly the most abundant, which may have a greater influence on forest fires in extreme conditions.

Due to the physicochemical properties of BVOCs, state-of-the-art analytical methodologies for monitoring include an enrichment step prior to gas chromatography coupled with mass spectrometry (GC-MS). In addition to active sampling techniques (e.g., purge and trap), passive sampling approaches have been proven to be much easier to operate and are in compliance with the green analytical chemistry (GAC) principles [3]. Modern passive sorption-based techniques, such as solid-phase microextraction (SPME), are the most successful enrichment techniques enabling the direct microextraction of the main volatile and semi-volatile compounds from several types of matrices through the headspace (HS) sampling mode while enhancing the very high selectivity and sensitivity. Apart from other technical possibilities, the HS-SPME is currently the most widely used referenced approach for BVOCs analysis, since it allows for the microextraction and concentration of analytes in a coating fiber in one single step prior to GC-MS analysis, and it is a solvent-free approach in line with the GAC principles. More recently, bar adsorptive microextraction (BAµE), a novel passive sorption-based technique, has been proposed for the trace analysis of target compounds with a wide range of polarity through the direct sampling mode in the liquid phase (e.g., wastewater, urine, etc.). Due to the very high selectivity and sensitivity of the different sorbent phases available, including some types of polymers (Ps) and activated carbons (ACs), this modern analytical technique has shown an effective performance for monitoring a large number of priority and emerging organic compounds, including herbicides, fungicides, pharmaceutical and personal care products and many other classes [4,5]. Furthermore, BAµE technology presents very interesting features, as it is a user- and eco-friendly approach as well as cost-effective, that make it unique and a real alternative compared to other common dedicated microextraction techniques. Meanwhile, BAµE was never proposed to be applied in the HS sampling mode for the analysis of volatile or semi-volatile organic compounds in order to demonstrate the great enrichment capacity and scope of this analytical tool.

**Figure 1:**
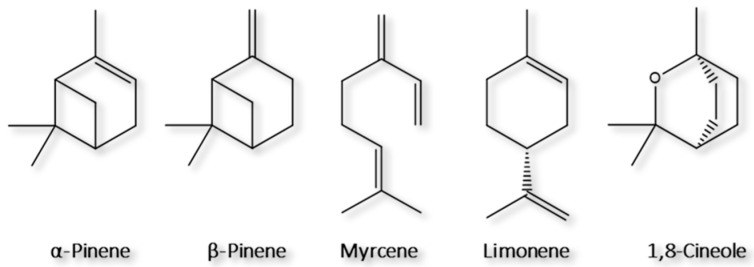
Chemical structures of the target monoterpenoids studied in the present work.

In the present contribution, HS-BAμE is proposed for the first time as an alternative passive microextraction technique for BVOCs analysis and is compared with the well-established HS-SPME technique. To evaluate the performance of both methodologies, the five major monoterpenoids (α-pinene, β-pinene, myrcene, limonene and 1,8-cineole; Figure 1) emitted by the leaves of the *Eucalyptus globulus* Labill. and *Pinus pinaster* Aiton tree species will be evaluated in combination with GC-MS analysis. The development, optimization, validation, and application to real matrices are also addressed, including the advantages and limitations regarding the analytical performance of both methodologies. The importance of monitoring BVOCs in natural forest environments to anticipate if the amounts founded may have an influence whenever forest fires occur under extreme conditions is also discussed.

## 2. Results and Discussion

### 2.1. GC-MS Instrumental Conditions

The development of the experimental procedure started with the improvement of the GC-MS parameters to establish the appropriate instrumental conditions for BVOCs analysis through both analytical methodologies. For such purpose, two standard mixtures of five BVOCs were analyzed by GC-MS using two inlet configurations; a 100 mg L^−1^ mix solution was analyzed in the split (S) injection mode (ratio of 1:40) for the HS-SPME/GC-MS methodology, and a 2 mg L^−1^ mix solution was analyzed in the splitless (SL) injection mode for the HS-BAμE/GC-MS approach. The total ion chromatograms obtained showed great sensitivity and symmetrical peak shapes for each compound with a suitable resolution under an acceptable analytical time (<10 min). The instrumental sensitivity was evaluated through the limits of detection (LODs) and quantification (LOQs) of each target compound by calculating the signal-to-noise (S/N) ratios of 3:1 and 10:1, respectively. When using the S injection mode, LODs and LOQs of 0.5 and 1.5 mg L^−1^ were obtained for the internal standard (IS), α-pinene and limonene, respectively. Meanwhile, for β-pinene, myrcene and 1,8-cineole, these analytical thresholds corresponded to 1.0 and 3.0 mg L^−1^, respectively. As for the SL injection mode injection, the LODs and LOQs achieved for all target compounds were 0.1 and 0.4 mg L^−1^, respectively. Afterwards, the instrumental precision was determined through six consecutive injections of the same standard mixtures (under the same injection conditions), obtaining values of the relative standard deviation (RSD) ≤ 10.6% for all analytes. The instrumental calibration was performed through the determination of linear regressions for each analyte, prepared with eleven concentration levels ranging from 20.0 to 700.0 mg L^−1^ (S mode, ratio of 1:40) and between 0.4 and 5.0 mg L^−1^ (SL mode), where remarkable linearity was attained, as all determination coefficient (*r*^2^) values were above 0.9963.

### 2.2. Development of the Microextraction Methodologies

**Figure 2:**
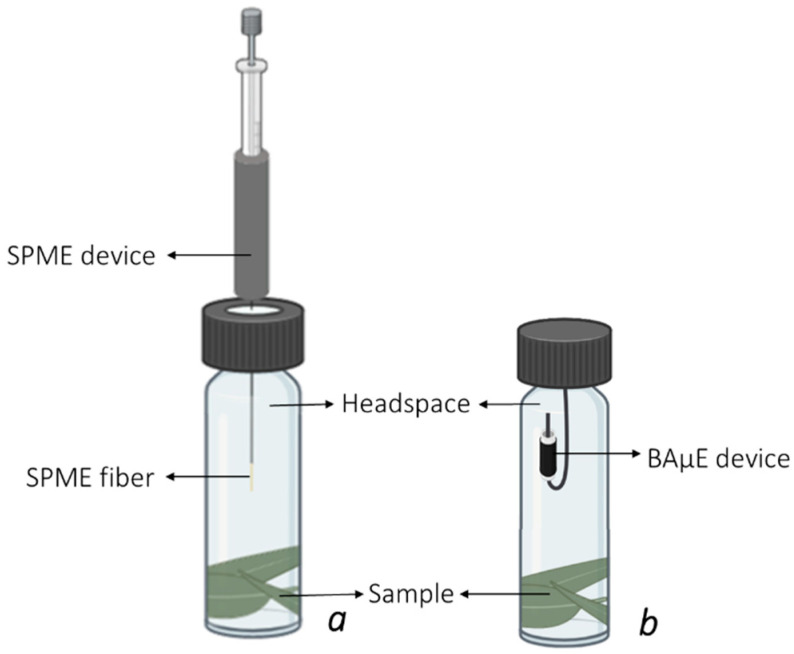
Experimental set-up for the HS-SPME (**a**) and HS-BAμE (**b**) assays.

After optimizing the instrumental system, the experimental conditions for the HS-SPME and HS-BAμE methodologies were developed and evaluated using the experimental set-up depicted in Figure 2.

#### 2.2.1. Optimization of the HS-SPME Methodology

**Figure 3:**
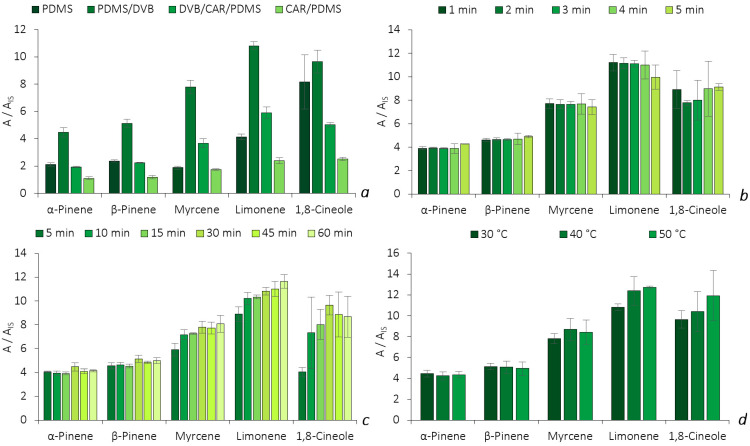
Effect of fiber coating selectivity (**a**), thermal desorption time (**b**), equilibrium time (**c**) and HS temperature (**d**) on the enrichment of the five BVOCs obtained by HS-SPME/GC-MS. The error bars represent the standard deviation of three replicates.

The assays were performed through HS analysis of the glass flasks spiked with a standard mixture of the five BVOCs. By using a one-variable-at-a-time (OVAT) strategy, different parameters were evaluated, including the fiber type, the thermal desorption time, the equilibrium time and the thermostatization temperature. The microextraction efficiency was assessed through the signal response, using the ratio of each individual area to the IS area (A/A_IS_). Figure 3 illustrates the microextraction efficiencies obtained for each parameter evaluated.

The optimization started with the selection of the best fiber coating (PDMS, PDMS/DVB, DVB/CAR/PDMS and CAR/PDMS) for the BVOCs analysis. All four SPME fibers extract through the sorption mechanism, where the analytes migrate to the coating phase through π-π, hydrogen, dipole–dipole, hydrophobic and ionic interactions. The retention of the compounds also depends on their physicochemical properties, as well as on the coating’s surface pore diameter [6]. In Figure 3a, the PDMS/DVB fiber showed a higher efficiency for the microextraction of all five BVOCs, as the response for the analytes was more than twice the response of the DVB/CAR/PDMS fiber and four times that of the CAR/PDMS fiber, which is in agreement with previous reports [7,8]. Due the higher selectivity achieved, the PDMS/DVB fiber was chosen for the further studies. Afterwards, we proceeded to optimize the thermal desorption time.

The results presented in Figure 3b show similar performances for both α- and β-pinene, in which a slightly higher response was obtained at 5 min. This was also verified with 1,8-cineole. Meanwhile, for limonene and myrcene, better results were obtained at 3 and 4 min, respectively. Given that the results obtained for the latter three BVOCs (limonene, myrcene and 1,8-cineole) at 5 min still ensured remarkable performance (7.4 ≤ A_i_/A_IS_ ≤ 10.0), it was selected as the thermal desorption time for the remaining optimization procedures. Additionally, the selection of a higher desorption time may also prevent cross-contamination issues between analytical runs, ensuring a good clean-up of the fiber.

Subsequently, we proceeded with the optimization of the equilibrium time. The SPME technique is a sorption-based microextraction approach, which means that the maximum amount of the analyte that the fiber can extract is achieved after reaching the equilibrium time [9]. As observed in Figure 3c, α-pinene, β-pinene, myrcene and 1,8-cineole show better recoveries at 30 min, while limonene does so after 60 min of extraction. Nevertheless, for limonene, the RSD value of the assays performed at 60 min (RSD = 5.0%) is two times higher than the one obtained at 30 min (2.5%), which indicates more dispersion between replicates and, consequently, lower precision. Hence, 30 min of equilibrium time was maintained for further experiments.

The last parameter of the optimization procedure included the assessment of the thermostatization temperature. The results showed that the HS microextraction efficiency of the procedure increases with the temperature for the monoterpenoids, except for β-pinene, where a slightly decrease occurs for the assays performed at temperatures higher than 40 °C (Figure 3d). An increase in the temperature directly affects the distribution of the analytes between the HS sampling mode and the fiber coating, as it increases the partition coefficient, which improves the capacity for extracting higher amounts. However, higher temperatures could also decrease the partition coefficient between the sample matrix and the fiber phase, leading to losses in efficiency and sensitivity [10]. In this sense, a temperature of 30 °C was selected, as it showed a lower variability in between replicates for all five monoterpenoids (RSDs ≤ 8.5%).

#### 2.2.2. Optimization of the HS-BAμE Methodology

**Figure 4:**
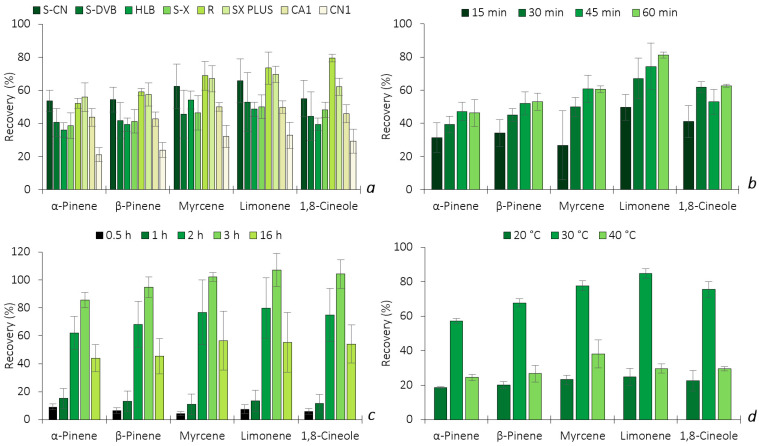
Effect of sorbent selectivity (**a**), LD time (**b**), equilibrium time (**c**) and temperature (**d**) on the enrichment of the five BVOCs obtained by HS-BAμE/GC-MS. The error bars represent the standard deviation of three replicates.

Analogously to HS-SPME, the optimization of HS-BAμE was also carried out, including the selection of the sorbent coating, the liquid desorption (LD) time, the solvent type, the equilibrium time and the temperature, regarding the enrichment of the five BVOCs. Starting with the sorbent phase selection, four Ps (S-CN, S-DVB, HLB and S-X) and four ACs (R, SX PLUS, CA1 and CN1) were tested. One of the great advantages of this technology is the possibility of tailoring or adapting the best coating phase for each particular type of application, using common and easily available sorbents. Figure 4a depicts the comparison of the recoveries for the five BVOCs by BAμE using eight different sorbents in the HS sampling mode. From the data obtained, it is possible to notice that the AC(R) sorbent presents the best recovery of all the target analytes, while the AC(SX PLUS) phase has a similar efficiency, with the exception of 1,8-cineole. The ACs are nanoporous solid materials that retain the BVOCs through electrostatic and/or dispersive interactions, according to the textural adsorptive properties, surface area, nanopore volume and dimensions, as well as the surface functional groups. Regarding the micropore network, from the studied ACs, only SX PLUS and R present both ultramicropores (≤0.7 nm) and supermicropores (0.7–2.0 nm), while CA1 and CN1 presented merely supermicropores (0.7–2.0 nm). Since the BVOCs studied are very small molecules, this might suggest that an AC with smaller pore sizes (i.e., ultramicropores) presents a better affinity to the target BVOCs, which results in higher microextraction efficiencies. Additionally, the surface chemistry may play a relevant role; the ACs SX PLUS and R present pH_PZC_ values of, respectively, 8.4 and 6.5, while the other ACs present clearly acidic surface chemistries (pH_PZC_ of 2.2 and 5.1). From the AC tested, R gathers the highest volume of ultramicropores and the narrower ultramicropores, as indicated by its high BET constant value (1666 versus 362–937 for the other ACs), associated with a pH_PZC_ value of 6.5. This combination of properties of R seems to enhance the performance of this AC for the quantification of the target BVOCs. In the case of Ps, the recoveries were all above 40%—in particular, P(S-CN) showed a similar response as AC(SX PLUS). Ps retained the target BVOCs through different mechanisms such as π-π, dipole–dipole, hydrogen bonds and ionic interactions, depending also on their surface area and particle size. From the results achieved, since AC(R) showed much higher selectivity and recovery yields, it was chosen as the sorbent coating to proceed with the further assays.

After selecting the most selective sorbent phase, the back-extraction optimization was carried out, including the solvent type and desorption time under ultrasonic conditions. Five solvents were tested (*n*-C_6_, DCM, MeOH, ACN and MeOH:ACN (1:1, *v*/*v*)) to obtain the best desorption removal. The results obtained (data not shown) prove that *n*-C_6_ was the only solvent that allowed for the complete back-extraction of all five BVOCs, while the remaining solvents did not present any signal during GC-MS analysis and, therefore, was selected for further experiments. The influence of the sonication time was also studied at 15, 30, 45 and 60 min. As shown in Figure 4b, the recoveries of α-pinene, β-pinene and myrcene were at the maximum at 45 min and 30 min for 1,8-cineole, while 60 min was needed for limonene, respectively. Since longer periods of desorption time do not decrease the efficiency of the other four BVOCs, 60 min was selected.

HS-BAμE is also a passive approach based on the equilibrium of analytes between the HS sampling mode and the sorbent phase, in which the equilibrium time always plays a key role during the microextraction process. The influence of this parameter was evaluated by carrying out experiments within 0.5, 1, 2, 3 and 16 h, as illustrated in Figure 4c, where the recovery yields increase until 3 h, whereas longer microextraction periods resulted in lower yields. These data could be explained by the fact that the less polar the compounds are, such as the target BVOCs (2.35 ≤ log *K*_O/W_ ≤ 3.54), the greater the adsorption on the inner walls of the sampling flasks is after longer periods of time, thus minimizing the efficiency [11]. Therefore, 3 h was chosen as the optimum equilibrium time and was used in the subsequent assays.

Finally, since this is the first time that BAμE has been applied in the HS sampling mode, the thermostatization can have a great influence on the efficiency, such as in the SPME technique, and, therefore, several assays were performed under different temperatures (20, 30 and 40 °C). According to Figure 4d, a temperature of 30 °C appears to be the optimum value in attaining the maximum microextraction recovery yields. Lower or higher temperatures seem to significantly decrease the efficiency performance due to the mass transfer equilibrium of the selected BVOCs towards the sorbent phase being strongly affected.

### 2.3. Validation and Comparison of Both Methodologies

After optimizing both methodologies (HS-SPME-fiber: PDMS/DVB, HS temperature: 30 °C, equilibrium time: 30 min and desorption time: 5 min; HS-BAµE-sorbent: AC(R), HS temperature: 30 °C, equilibrium time: 3 h and desorption solvent: *n*-C_6_), validation assays were performed, including analytical thresholds, linear dynamic ranges, precision and accuracy. Table 1 summarizes the analytical data obtained from the parameters evaluated during the validation assays for both methodologies, under optimized experimental conditions.

Analytical thresholds were evaluated through the determination of the LODs and LOQs, in which the values achieved were in the ranges of 25.0–50.0 ng L^−1^ and 75.0–175.0 ng L^−1^ for HS-SPME(PDMS/DVB), whereas 5.0 µg L^−1^ and 16.6 µg L^−1^ were obtained for HS-BAµE(R), respectively (Table 1). Nevertheless, this difference is not surprising according to the analytical configuration adopted, since HS-SPME resorts to an approach in which the matrix sampled in the fiber is fully analyzed (‘one-shot analysis’) by thermal desorption (TD), reflected by the lowest LODs achieved, whereas the HS-BAµE relies on LD, where only a portion (1 µL) is injected into the GC-MS system.

The linear dynamic ranges were found through the analysis of the standard mix solutions of the five monoterpenoids ranging from 0.5 to 17.5 µg L^−1^ (HS-SPME(PDMS/DVB)) and between 20.0 and 100.0 mg L^−1^ (HS-BAμE(R)), where remarkable linearity was found (*r*^2^ ≥ 0.9959). Nevertheless, the *r*^2^ is not enough to verify the linearity of the regressions, since the evaluation of the heteroscedasticity data remains unassessed. In this sense, the homoscedasticity data were evaluated through the lack-of-fit test and residues dispersion [12]. For the conventional linear regressions plotted, the lack-of-fit test showed a good fit (at a confidence level of 95%) of the calibration curves (i.e., F_exp_ < F_tab_), suggesting a homoscedastic distribution of the experimental data. Moreover, through the study of the residue plots, an unsystematic distribution of the relative residues was verified, which confirms the homoscedasticity of the experimental data, as well as the suitability of the linear regressions plotted for the analyses of the five monoterpenoids. Furthermore, the lower limit of quantification (LLOQ) of the five BVOCs was determined for both analytical methodologies. The LLOQ corresponds to the lowest concentration level, which might be quantified under both precision and accuracy criteria (RSD and %RE ≤ 20 %) and normally corresponds to the first value of the calibration plots [13,14].

As noted in Table 1, LLOQs values of 0.5 µg L^−1^ (HS-SPME(PDMS/DVB)) and 20 mg L^−1^ (HS-BAμE(R)) were achieved for the five BVOCs, respectively. Moreover, the slopes from the regression plots obtained through the HS-SPME(PDMS/DVB) methodology (26.193 ≤ *a* ≤ 75.885) are significantly higher than the ones obtained by HS-BAμE(R) (4.508 ≤ *a* ≤ 7.184); we can establish that the former presents a much higher sensitivity, using the analytical configuration conditions reported. The accuracy and precision of both methodologies were evaluated through the determination of the percentage of the relative error (%RE) and RSD values, respectively, through intra-day (*n* = 6) and inter-day (*n* = 6, over 3 consecutive days) assays. In both repeatability assessments, four different spiking levels were evaluated, including the LLOQ and a low, medium and higher value. For the HS-SPME(PDMS/DVB) methodology, levels of 20, 100, 400 and 700 ng were selected, whereas for the HS-BAμE(R) approach, the levels were 100, 200, 300 and 500 ng. Through both intra-day and inter-day studies, the analytical methodologies exhibited good results at the LLOQ (RSD and %RE ≤ 19.9%) and other spiking levels (RSD and %RE ≤ 14.2%), which indicates a good fit for the assessment of the five selected BVOCs in the HS sampling mode.

In general, we can state that both analytical methodologies present a very similar effectiveness for BVOCs analysis, since excellent reproducibility is achieved in monitoring the five monoterpenoids under the HS sampling mode. If we assume that HS-SPME is a well-established standard or reference method, whereas HS-BAμE is a novel proposed approach for BVOCs analysis, we can claim that both methodologies are equivalent, providing similar and proportional responses.

**Figure 5:**
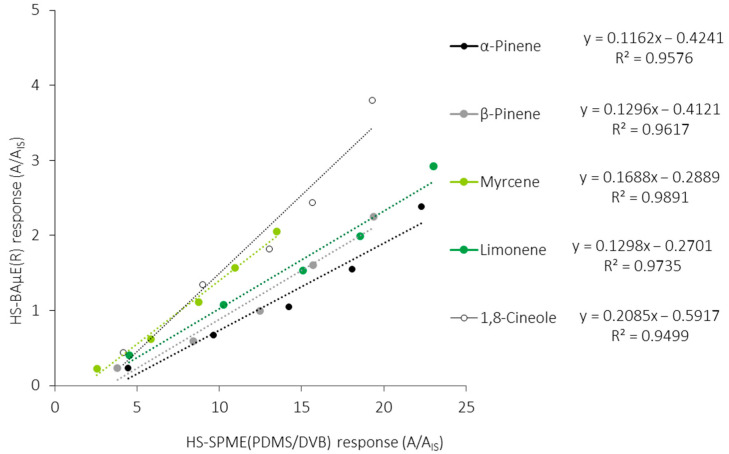
Comparison of the response of HS-BAμE(R) against the HS-SPME(PDMS/DVB) methodology, followed by GC-MS analysis, for the five major monoterpenoids, under optimized experimental conditions.

Figure 5 shows the comparison data obtained from the HS-BAµE(R) response against the HS-SPME(PDMS/DVB) response for the five monoterpenoids, under optimized experimental conditions, where it is verified that the latter methodology presents an average order of magnitude between five (1,8-cineole) and ten (α-pinene) times higher, which is reflected by the slopes founded. It can also be observed that the different responses obtained for each monoterpene depend on the best interaction and affinity with the sorbent phases used, as well as the synergism involved simultaneously among the target compounds involved. Moreover, if we assume that both analytical technologies can be routinely used and are in line with the GAC principles, or that the inherent aspects are negligible, we can state that the HS-BAµE methodology is a much more cost-effective and comprehensive technique, since it can be easily interfaced with different instrumental systems. On the other hand, from a strictly practical point of view, the HS-SPME methodology, based on TD, seems to be more user-friendly, whereas HS-BAµE, based on LD, offers the advantage of being able to carry out a reanalysis of the sampled matrices, which is a very important issue whenever the repetition and data confirmation are mandatory.

### 2.4. Application to Real Samples

**Figure 6:**
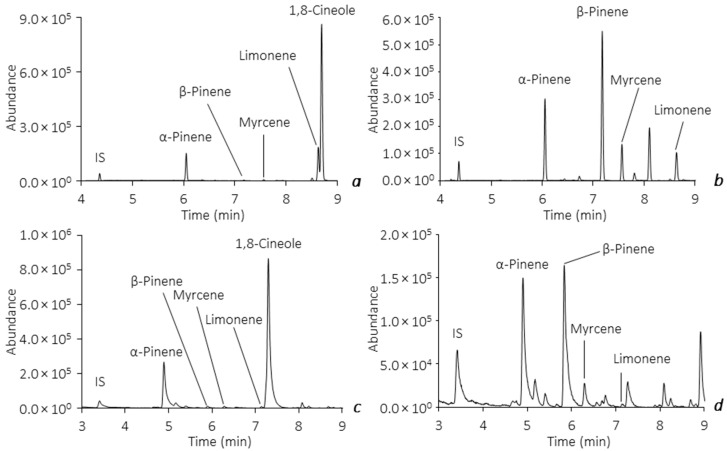
Example of total ion chromatograms obtained from assays on the leaves of *E. globulus* Labill. and *P. pinaster* Aiton by HS-SPME(PDMS/DVB)/GC-MS (**a**,**b**) and HS-BAμE(R)/GC-MS (**c**,**d**), respectively, under optimized experimental conditions.

Both of the optimized and validated analytical methodologies were subsequently applied in the analysis of BVOCs released by the leaves from the *E. globulus* Labill. and *P. pinaster* Aiton species, sampled in ‘Parques de Sintra’ during June 2020 and September 2021. The main goal of this survey was to evaluate the average number of the major monoterpenoids emitted from the leaves and, therefore, anticipate the influence that they may have in the natural forest environment, particularly under the occurrence of forest fires. Assays performed on the BVOCs release from the leaves of the tree species showed, as expected, identical pattern profiling through both methodologies, demonstrating similar analytical responses, as stated before. Figure 6 exemplifies the total ion chromatograms obtained from in vitro assays on the leaves of *E. globulus* Labill. and *P. pinaster* Aiton by the HS-SPME(PDMS/DVB)/GC-MS (a,b) and HS-BAμE(R)/GC-MS (c,d) methodologies, respectively, in which identical profiles are achieved, as well as remarkable selectivity and sensitivity. During the aforementioned sampling period, the average levels obtained from assays on the major BVOCs released by the leaf samples indicated that *P. pinaster* Aiton presented very significant emissions of β-pinene (3503.8 ± 396.3–159.4 ± 19.1 μg g^−1^), α -pinene (1281.6 ± 168.5–105.7 ± 1.6 μg g^−1^), limonene (731.9 ± 115.3–9.2 ± 1.4 μg g^−1^) and myrcene (397.7 ± 28.9–10.3 ± 0.8 μg g^−1^). For the leaf samples from *E. globulus* Labill., considerable emissions of the major BVOCs were also observed (95.2 ± 15.6 μg g^−1^ < α-pinene < 3183.3 ± 353.6 μg g^−1^; 39.6 ± 1.5 μg g^−1^ < limonene < 883.0 ± 18.4 μg g^−1^; 18.4 ± 1.2 μg g^−1^ < myrcene < 131.6 ± 14.1 μg g^−1^, 10.2 ± 1.3 μg g^−1^ < β-pinene < 78.2 ± 11.8 μg g^−1^), and, as expected, 1,8-cineole was only detected in this species in very significant amounts (7828.0 ± 40.0–670.7 ± 110.8 μg g^−1^).

Nevertheless, the maximum contents detected for α-pinene, β-pinene and myrcene were lower compared with those reported by Sampetro et al. [15], who reported amounts of 4357.5, 6187.2 and 1377.8 μg g^−1^, respectively, from the *P. pinaster* Aiton species by using a conventional analytical approach, constituted by ultrasonic extraction with organic solvents prior to GC-MS analysis. The contents detected in the present study for limonene were relatively higher than the reported value (463.2 μg g^−1^).

Furthermore, the concentrations variability on the major BVOCs of the same tree species is probably associated with the different chemotypes, which could affect the pattern of compounds released [16]. From the data achieved, we can state that there was a clear variation in the BVOCs emission over the period of time in which the leaf sampling took place, although it was not possible to quite understand the pattern that could explain the reason for such variability, whether from biological, atmospheric (temperature, moisture, wind, etc.) or anthropogenic constraints that can promote stressful conditions for the tree species or any other reason. However, the number of volatiles emitted by the sampled leaves is very significant and, therefore, may contribute to the accumulation of BVOCs in the natural forest environment, under certain atmospheric conditions. In general, at 30 °C, the maximum concentrations emitted from these BVOCs by the crowns of the tree species under study (0.01–1.26 g m^−3^) are far below their lower flammability limits (LFL), which, for 1,8-cineole, is between 38.2 and 38.8 g m^−3^. As higher temperatures lead to increasing BVOCs emissions, a preliminary study comprising the variation of the 1,8-cineole concentration emitted by the tree crowns of *E. globulus* Labill. suggests that its LFL can be easily exceeded at 138 °C and, consequently, might act as fuel for the forest fire’s propagation, particularly under extreme weather conditions (temperature: >30 °C; wind: >30 km/h; moisture: <30%).

## 3. Materials and Methods

### 3.1. Chemical Standards, Material and Real Samples

Acetonitrile (ACN, CH_3_CN, 99.9%), dichloromethane (DCM, CH_2_Cl_2_, 99.9%), *n*-hexane (*n*-C_6_, C_6_H_14_, 96%), *n*-pentane (C_5_H_12_, 95%) and *n*-heptane (C_7_H_16_, 99%), all of HPLC-grade, were obtained from Carlo Erba (Peypin, France); meanwhile, HPLC-grade methanol (MeOH, CH_3_OH, 99.9%) was acquired from Honeywell (Saint-Germain-en-Laye, France); *n*-nonane (C_9_H_20_, 99%) and a standard mixture of *n*-alkanes C_10_–C_40_ (50 mg L^−1^ of each in *n*-heptane) were purchased from Fluka (St. Gallen, Switzerland). Standards of (+)-α-pinene (α-pinene; C_10_H_16_; 98%; St. Louis, MO, USA), (−)-β-pinene (β-pinene; C_10_H_16_; 99%; St. Louis, MO, USA), 1,8-cineole (C_10_H_18_O; 99%; St. Louis, MO, USA), myrcene (C_10_H_16_; technical grade; Saint-Quentin, France), *R*-(+)-limonene (limonene; C_10_H_16_; 97%; Toluca, Mexico) and bromopentafluorobenzene (used as an internal standard, IS; C_6_BrF_5_; 99%; Gillingham, UK) were purchased from Sigma-Aldrich. The stock solutions of the individual analytes and IS were prepared by dissolving them in *n*-C_6_ to obtain a final concentration of 5000 mg L^−1^ and 1000 mg L^−1^, respectively. Ultra-pure water was obtained from a Milli-Q water purification system from Merck Millipore (Burlington, MA, EUA). The solutions were stored in glass flasks at −20 °C and renewed every month. Working standard mixtures (1000 mg L^−1^) and IS (20 mg L^−1^) were prepared daily in *n*-C_6_ by appropriate dilution from stock solutions. For the HS-SPME assays, a manual holder was used, and the fibers tested were polydimethylsiloxane (PDMS, 100 μm), polydimethylsiloxane–divinylbenzene (PDMS/DVB, 65 μm), Carboxen^TM^–polydimethylsiloxane (CAR/PDMS, 75 μm) and divinylbenzene–Carboxen^TM^–polydimethylsiloxane StableFlex^TM^ (DVB/CAR/PDMS, 50/30μm), which were all obtained from Supelco^®^ (Bellefonte, PA, USA). The sorbent phases tested for BAμE devices included four Ps, namely, the reverse phase *N*-vinylpirrolidone polymer Strata^TM^ X (S-X; particle size: 33 μm; pore size: 85 Å, surface area (A_BET_): 800 m^2^ g^−1^), the reverse phase styrene-divinylbenzene co-polymer Strata^®^ SDB-L (S-DVB; particle size: 100 μm; pore size: 280 Å, surface area (A_BET_): 500 m^2^ g^−1^) and the cyano-based co-polymer Strata^®^ CN (S-CN; particle size: 55 μm; pore size: 70 Å, surface area (A_BET_): 500 m^2^ g^−1^), all supplied from Phenomenex (Torrance, CA, USA), as well as the reverse phase *N*-vinylpirrolidone-divinylbenzene co-polymer Oasis^®^ HLB (HLB; particle size: 30 μm; pore size: 80 Å, surface area (A_BET_): 800 m^2^ g^−1^) from Waters (Milford, MA, USA). The four commercial ACs used included the CA1 (pH_PZC_: 2.2; surface area (*A*_BET_): 1043 m^2^ g^−1^; *V*_mesopores_: 0.66 cm^3^ g^−1^; *V*_α supermicropores_: 0.26 cm^3^ g^−1^), the CN1 (pH_PZC_: 5.1; surface area (*A*_BET_): 1179 m^2^ g^−1^; *V*_mesopores_: 0.68 cm^3^ g^−1^; *V*_α supermicropores_: 0.30 cm^3^ g^−1^) and the SX PLUS (pH_PZC_: 8.4; surface area (*A*_BET_): 833 m^2^ g^−1^; *V*_mesopores_: 0.44 cm^3^ g^−1^; *V*_α supermicropores_: 0.15 cm^3^ g^−1^; *V*_α ultramicropores_: 0.08 cm^3^ g^−1^) from Salmon & Cia (Portugal) and the R (pH_PZC_: 6.5; surface area (*A*_BET_): 964 m^2^ g^−1^; *V*_mesopores_: 0.40 cm^3^ g^−1^; *V*_α supermicropores_: 0.16 cm^3^ g^−1^; *V*_α ultramicropores_: 0.09 cm^3^ g^−1^) from Riëdel-de Haën (Hannover, Germany). The textural and surface properties of the commercial ACs were assessed according to the procedures described by Ahmad et al. [17].

The *E. globulus* Labill. and *P. pinaster* Aiton leaves were sampled biweekly from June 2020 to September 2021 in ‘Parques de Sintra’ (Monte da Lua and Monserrate) in the Lisbon region (Portugal), with a total of 132 samples. Fresh leaves were cut and stored in a sealed glass bottle within a thermal box. Upon arrival to the laboratory, all leaves were stored at −20 °C until analysis.

### 3.2. Experimental Set-Up

#### 3.2.1. Preparation and Conditioning of SPME Fibers and BAμE Devices

Each SPME fiber was thermally conditioned according to the manufacturer’s recommendation conditions before each microextraction: 30 min at 250 °C (PDMS and PDMS/DVB), 30 min at 270 °C (CAR/PDMS) and 60 min at 300 °C (DVB/CAR/PDMS). The BAμE devices were ‘lab-made’ by using polypropylene subtracts (7.5 × 3.0 mm) coated first with a suitable adhesive film followed by the fixation of different powdered sorbents (Ps and ACs) [13,18,19]. The coated BAμE devices were then cleaned up in ultrapure water and dried out with a paper towel prior to the enrichment step to remove any possible remaining impurities.

#### 3.2.2. Experimental Optimization Conditions (HS-SPME and HS-BAμE Assays)

To optimize the HS-SPME experimental conditions, 1 μL of a mix solution of the five selected BVOCs (100.0 mg L^−1^) and IS (20.0 mg L^−1^) was spiked at the bottom of 40 mL glass flasks, which resulted in final masses of 100 and 20 ng for each VOC and IS, respectively. The flasks were then closed with septum caps suitable for the HS analysis and preconditioned in a thermostatic sand bath (30 min) at the selected temperature. The microextraction was performed by exposing the SPME fiber to the HS of the flasks (Figure 2a). After the microextraction, the SPME fiber was collected in the device and removed from the sample flasks, it being immediately inserted into and exposed to the GC-MS injection port for the thermal desorption (TD) and direct analysis of the extracted BVOCs. All analyses were performed in triplicate. Blank assays without spiking were also performed to assess the absence of analyte transference and possible carry-over. The approach used for the optimization of the diverse experimental parameters consisted in a one-variable-at-a-time (OVAT) strategy; while the study of the behavior of one variable was being performed, all the remaining were fixed. The selected parameters for the optimization process of the HS-SPME methodology were SPME fiber material (PDMS, PDMS/DVB, CAR/PDMS and DVB/CAR/PDMS), thermal desorption time (1, 2, 3, 4 and 5 min), equilibrium time (5, 10, 15, 30, 45 and 60 min) and thermostatization temperature (30, 40 and 50 °C), in accordance with previous reports [7,9,10,13,14,20,21,22].

For the optimization of the HS-BAμE experimental conditions, 100 μL of a mix solution containing the five selected BVOCs (5.0 mg L^−1^) was spiked at the bottom of 5 mL glass flasks, which corresponds to a total of 500 ng of each. The previously conditioned BAμE devices were placed into the HS of the flasks (Figure 2b), which were immediately closed up and placed in a thermostatic sand bath for the enrichment step. After the microextraction, the BAμE devices were removed from the glass flasks with clean tweezers and transferred into glass vials with inserts containing 90 μL of an organic solvent; then, they were subjected to an ultrasonic treatment (Branson 3510 E-DTH, 42 +/− 2.5 kHz, 100 W, Switzerland) for liquid desorption (LD). Subsequently, 10 μL of a 20 mg L^−1^ IS solution was added into the glass inserts, resulting in a final concentration of 2 mg L^−1^. Finally, the BAμE devices were removed from the vial, which were sealed for GC-MS analysis. Each assay and blank were performed in triplicate. The optimization of each experimental parameter was performed in a similar manner as the HS-SPME optimization, i.e., a one-variable-at-a-time (OVAT) strategy. The selected parameters for the optimization process of the HS-BAμE were the sorbent coating material (ACs: CA1, CN1, SX PLUS and R; Ps: S-X, S-DVB, S-CN and HLB), desorption solvent (*n*-C_6_, DCM, MeOH and ACN), desorption time (15, 30, 45 and 60 min), equilibrium time (0.5, 1, 2, 3 and 16 h) and thermostatization temperature (20, 30 and 40 °C), according to previous works [5,13,18,19,23,24]. The data obtained by the HS-BAμE/GC-MS studies were determined by comparing the signal areas with the standard solutions from all five target BVOCs.

#### 3.2.3. Validation Assays

For validation purposes, several assays were performed for both analytical methodologies under optimized experimental conditions. Two experimental configurations were adopted; for the HS-SPME(PDMS/DVB)/GC-MS, 1 μL of the BVOCs mix solution (with the desired concentration) and IS (20.0 mg L^−1^) were added into 40 mL glass flasks; for the HS-BAμE(R)/GC-MS, 100 μL of the BVOCs mix solution was spiked into 5 mL glass flasks, using the same procedures as in the previous section. The validation process of both methodologies involved the assessment of several parameters, including linearity, sensitivity, selectivity, accuracy and precision, according to previous studies [5,7,9,13,14,18,19,23,24,25]. Initially, the selectivity of both analytical methodologies was evaluated by verifying the absence of interfering compounds with identical retention times for each analyte, performed by means of both HS-SPME/GC-MS and HS-BAμE/GC-MS analyses of blank assays. Afterwards, calibration curves were plotted by analyzing the calibration standards of the five BVOCs in concentrations ranging from 20.0 to 700.0 mg L^−1^ for HS-SPME(PDMS/DVB)/GC-MS (equivalent to total masses of 20 and 700 ng, respectively) and between 1.0 and 5.0 mg L^−1^ for HS-BAμE(R)/GC-MS (equivalent to total masses of 100 and 500 ng, respectively). The linearity was then assessed through the lack-of-fit test, as well as the verification of the residual dispersion and determination coefficient (*r*^2^) of each calibration plot [26]. The sensitivity of both methodologies was evaluated by determining the limit of detection (LOD), limit of quantification (LOQ) and lower limit of quantification (LLOQ). The LOD and LOQ correspond to signal-to-noise (S/N) values of 3/1 and 10/1, respectively, while the LLOQ is the lower concentration, which is measurable according to accuracy and precision acceptance criteria and usually used as the first point of the calibration plot. To assess intra-day (*n* = 6) as well as inter-day (*n* = 6, during 3 consecutive days) accuracy and precision, assays were performed under optimized conditions for the analysis of the HS of the glass flasks after spiking with the five BVOCs and the IS mix solution. For the HS-SPME(PDMS/DVB)/GC-MS analytical approach, the IS concentration was maintained at 20 mg L^−1^ (equivalent to 20 ng); meanwhile, four spiking levels of the analyte were evaluated: the LLOQ, followed by a low, medium and high concentration, equivalent to masses of 20, 100, 400 and 700 ng, respectively, from each VOC; for the HS-BAμE(R)/GC-MS assays, the IS concentration was 2 mg L^−1^ (equivalent to 20 ng); meanwhile, the selected four concentrations for the BVOCs mix were equivalent to 100, 200, 300 and 500 ng. The acceptance criteria used for accuracy and precision were the percentage of the relative error (%RE) and the relative standard deviations (RSDs), which were ≤ 15 % for all spiking levels, except at the LLOQ, where values of RSD and RE ≤ 20 % were accepted [13,26]. Whenever indicated, all validation assays were performed in triplicate.

#### 3.2.4. Real Samples Assays

To evaluate the performances of the HS-SPME(PDMS/DVB) and HS-BAμE(R) methodologies on real matrices, BVOCs analysis was performed on the sampled leaves (Figure 2a,b). Before each analysis, fresh leaves of *E. globulus* Labill. and needles from *P. pinaster* Aiton trees were cut and weighed in 40 mL glass flasks, using amounts of 2 and 5 mg, respectively, and for the HS-SPME(PDMS/DVB) assays, 1 μL of the IS solution (20 mg L^−1^) was introduced into the flask, which was conditioned over 30 min (30 °C), followed by HS extraction under previously optimized experimental conditions. Assays were performed in duplicate for the leaves of each tree species from each sampling. The amounts of α-pinene, β-pinene, myrcene, limonene and 1,8-cineole emitted by the leaves of both tree species were determined through the previously obtained calibration plots. For quantification purposes, the dry weight (DW) leaves were determined by volatilization gravimetry. Thus, fresh leaves from all samplings were weighed to determine their fresh weight (FW) and then dried in a previously heated oven (80 °C) until their weight was constant (approximately 48 h) to eliminate the leaf moisture [27]. This allowed for the determination of the mass of each compound released by DW leaves (μg g^−1^).

### 3.3. Instrumental Set-Up

GC-MS analyses were performed on an Agilent 6890 series gas chromatograph equipped with an Agilent 7683 automatic liquid sampler and interfaced to an Agilent 5973N mass selective detector (Agilent Technologies, Little Falls, DE, USA). A split/splitless (S/SL) injector operating at 250 °C was used in two inlet configurations: manual injection in the S mode (ratio of 1:40) for the HS-SPME/GC-MS assays; automatic injection in the SL mode (injecting a volume of 1 μL) for the HS-BAμE/GC-MS assays. A fused silica capillary column (30 m length × 0.25 mm I.D. × 0.25 μm film thickness (Zebron ZB-5; 5% diphenyl, 95% dimethyl polysiloxane; Phenomenex, Torrance, CA, USA)), was used. The oven temperature was programmed to start at 50 °C followed by a 1 min isothermal, and then heated up to 90 °C at 5 °C min^−1^ (held isothermally for 1 min), heated up to 200 °C (20 °C min^−1^, held for 2 min) and, finally, heated up to 300 °C (25 °C min^−1^, held for 5 min), which led to a total run time of 27 min. Helium was used as the carrier gas in the constant pressure mode (14.3 psi). For all analyses a solvent delay of 3.5 min was used. The transfer line, ion source and quadrupole temperatures were maintained at 280 °C, 230 °C and 150 °C, respectively. In the full-scan mode, electron ionization mass spectra in the range of 45–550 Da were recorded at 70 eV of energy. The acquisition data and instrument control were performed by the MSD ChemStation software (G1701CA, version E.02.02.1431; Agilent Technologies, Santa Clara, CA, USA). The identity of each compound was assigned by the comparison of their retention indices (RI) relative to a mixture of C_10_–C_40_ *n*-alkane homologs [28] and the characteristic features of their mass spectra in comparison with the Wiley’s library spectral data bank (G1035B, Rev D.02.00; Agilent Technologies, Santa Clara, CA, USA). The calculations of each assay were performed by a comparison of the average peak areas of the extracted compounds with the IS peak area.

## 4. Conclusions

A novel analytical approach is proposed (HS-BAμE) in combination with GC-MS for monitoring VOCs. The methodology was fully developed, optimized and applied for the analysis of five BVOCs (α-pinene, β-pinene, myrcene, limonene and 1,8-cineole) and compared with the well-established HS-SPME, which is commonly accepted as a reference technique. The data obtained demonstrate that the new analytical approach (HS-BAμE(R)/GC-MS) exhibits great performance and robustness and is equivalent to HS-SPME(PDMS/DVB)/GC-MS, providing similar profiling and proportional responses, according to the analytical configuration used. Assays performed on the real matrices of leaves from *E. globulus* Labill. and *P. pinaster* Aiton by both analytical technologies showed high selectivity and sensitivity, with significant emissions of the major monoterpenoids.

This is the first time that the BAμE technology was applied in the HS sampling mode, and, in addition to other advantages, it proved to be a promising analytical alternative for VOC monitoring, given its great simplicity, easy handling and low cost.

## Figures and Tables

**Table 1:** LODs, LOQs, linear dynamic ranges, slopes (*a*), x-axis interceptions (*b*) and determination coefficients (*r*^2^) values from the calibration plots obtained for the five monoterpenoids by HS-SPME(PDMS/DVB) and HS-BAμE(R), followed by GC-MS analysis, under optimized experimental conditions.

Monoterpenoids	HS-SPME(PDMS/DVB)						HS-BAμE(R)					
	LOD (ng L^−1^)	LOQ (ng L^−1^)	Linear Range (µg L^−1^)	*a*	*b*	*r* ^2^	LOD (µg L^−1^)	LOQ (µg L^−1^)	Linear Range (mg L^−1^)	*a*	*b*	*r* ^2^
α-Pinene	25.0	75.0	0.5–17.5	26.193	−0.029	0.9987	5.0	16.6	20.0–100.0	4.731	−0.276	0.9976
β-Pinene	50.0	175.0		32.305	−0.047	0.9984				4.734	−0.296	0.9991
Myrcene	50.0	175.0		51.874	−0.480	0.9985				4.508	−0.256	0.9959
Limonene	25.0	75.0		75.885	−0.324	0.9989				5.630	−0.201	0.9965
1,8-Cineole	25.0	175.0		54.062	0.581	0.9966				7.184	−0.362	0.9976

## Data Availability

Not applicable.

## Sample Availability

Not available.
